# The Significance of EEG Alpha Oscillation Spectral Power and Beta Oscillation Phase Synchronization for Diagnosing Probable Alzheimer Disease

**DOI:** 10.3389/fnagi.2021.631587

**Published:** 2021-06-07

**Authors:** Haifeng Zhang, Xinling Geng, Yuanyuan Wang, Yanjun Guo, Ya Gao, Shouzi Zhang, Wenjin Du, Lixin Liu, Mingyan Sun, Fubin Jiao, Fang Yi, Xiaoli Li, Luning Wang

**Affiliations:** ^1^Medical School of Chinese People’s Liberation Army, Beijing, China; ^2^Department of Neurology, The 2nd Medical Center, National Clinical Research Center for Geriatric Disease, Chinese People’s Liberation Army General Hospital, Beijing, China; ^3^Health Service Department of the Guard Bureau of the Joint Staff Department, Joint Staff of the Central Military Commission of Chinese PLA, Beijing, China; ^4^School of Biomedical Engineering, Capital Medical University, Beijing, China; ^5^Department of Neurology, Beijing Tongren Hospital, Capital Medical University, Beijing, China; ^6^Department of Geriatrics, The Second Hospital of Hebei Medical University, Shijiazhuang, China; ^7^The Psycho Department of Beijing Geriatric Hospital, Beijing, China; ^8^Department of Neurology, Air Force Medical Center, Chinese People’s Liberation Army, Beijing, China; ^9^Ninth Health Care Department of the Second Medical Center of PLA General Hospital, Beijing, China; ^10^Department of Neurology, Lishilu Outpatient, Jingzhong Medical District, Chinese People’s Liberation Army General Hospital, Beijing, China; ^11^State Key Laboratory of Cognitive Neuroscience and Learning, Beijing Normal University, Beijing, China

**Keywords:** Alzheimer disease, electroencephalogram (EEG), power spectrum, spectral entropy (SE), phase synchronization index

## Abstract

Alzheimer disease (AD) is the most common cause of dementia in geriatric population. At present, no effective treatments exist to reverse the progress of AD, however, early diagnosis and intervention might delay its progression. The search for biomarkers with good safety, repeatable detection, reliable sensitivity and community application is necessary for AD screening and early diagnosis and timely intervention. Electroencephalogram (EEG) examination is a non-invasive, quantitative, reproducible, and cost-effective technique which is suitable for screening large population for possible AD. The power spectrum, complexity and synchronization characteristics of EEG waveforms in AD patients have distinct deviation from normal elderly, indicating these EEG features can be a promising candidate biomarker of AD. However, current reported deviation results are inconsistent, possibly due to multiple factors such as diagnostic criteria, sample sizes and the use of different computational measures. In this study, we collected two neurological tests scores (MMSE and MoCA) and the resting-state EEG of 30 normal control elderly subjects (NC group) and 30 probable AD patients confirmed by Pittsburgh compound B positron emission tomography (PiB-PET) inspection (AD group). We calculated the power spectrum, spectral entropy and phase synchronization index features of these two groups’ EEG at left/right frontal, temporal, central and occipital brain regions in 4 frequency bands: δ oscillation (1–4 Hz), θ oscillation (4–8 Hz), α oscillation (8–13 Hz), and β oscillation (13–30 Hz). In most brain areas, we found that the AD group had significant differences compared to NC group: (1) decreased α oscillation power and increased θ oscillation power; (2) decreased spectral entropy in α oscillation and elevated spectral entropy in β oscillation; and (3) decrease phase synchronization index in δ, θ, and β oscillation. We also found that α oscillation spectral power and β oscillation phase synchronization index correlated well with the MMSE/MoCA test scores in AD groups. Our study suggests that these two EEG features might be useful metrics for population screening of probable AD patients.

## Introduction

Alzheimer disease (AD) is the most common cause of dementia, accounting for an estimated 60–80% of cases (Garre-Olmo, 2018). It is characterized by progressive decline in memory, language function, orientation, and executive function, etc. AD is a continuous disease process, divided into preclinical, prodromal, and overt dementia (Babiloni et al., 2020a). Besides seriously affecting patients’ own quality of life, AD also brings heavy economic and psychological burdens to family members and caregivers, and has become one of the serious public health problems. The exact pathogenesis of AD is unclear yet; its related pathological hypotheses may involve synapse damage and loss, amyloid plaques and neurofibrillary tangles (Colom-Cadena et al., 2020). The pathophysiological process of AD is thought to start up to 20 years before clinical symptoms can be detectable (Sperling et al., 2014). At present, no effective medication exist for curing this pathology and reversing the course of AD (Cassani et al., 2018). Current therapeutic treatments at the early stage might improve the symptoms and delay the evolution of the disease (Houmani et al., 2018). Therefore, early diagnosis and active intervention are of great significance for mitigating the epidemic.

Current diagnosis of AD usually depends on the biomarkers in cerebrospinal fluid (CSF), neuropsychological tests, and neuroimaging, neurophysiological examinations (Maestu et al., 2019). The CSF biomarker like amyloid-β (Aβ) or tau protein level has high sensitivity and specificity in diagnosing probable AD (Lim et al., 2016). But the CSF biomarker is obtained from invasive lumber puncture operation, which is not easy to be accepted by patients and their families. Neuroimaging biomarker like the Pittsburgh compound B positron emission tomography (PiB-PET) inspection is highly specific in detecting the accumulation of in vivo amyloid-β, making it almost comparable to the golden standard of autopsy (Cohen et al., 2019). However, the PET inspection is very expensive, and it requires complex hardware equipment, inspection environment, and repeat exposure to radiation. Therefore, the above-mentioned two well-established biomarkers are not suitable for large-scale population screening. On the other hand, the neuropsychological test like the Mini-Mental Status Exam (MMSE) and Montreal Cognitive Assessment (MoCA) is easy to perform and can quickly evaluate a patient’s cognitive function. Therefore, these neurological tests are frequently used in clinical practice for screening large populations of possible AD. However, performing these tests is time-consuming, requires well-cooperated subjects and experienced clinicians (Cassani et al., 2018). Even though, the test scores are very subjective, and usually affected by the educational background of subjects.

As a non-invasive, cost-effective electrophysiological examination technique, electroencephalogram (EEG) can directly record the neural activity in different brain states. It can objectively and quantitatively reflect the neurological changes in pathological conditions with high time resolution, although its spatial resolution is lower than neuroimaging devices like magnetic resonance imaging (MRI). EEG has been widely used in the study of various neurological diseases including AD. Recently, Babiloni and many researchers have proposed an international initiative to include the use of EEG/MEG biomarkers in the regulatory requirements and guidelines for AD studies (Babiloni et al., 2020a). A variety of quantitative analysis techniques was used to characterize the EEG changes, looking for EEG biomarkers suitable for AD diagnosis. Compared to normal elderly, the resting state EEG activity in AD patients diffusely slows down, usually manifested by a decrease in the spectral power of the high frequency (α and/or β) oscillations and an increase of spectral power of low frequency (θ and/or δ) oscillations (Jeong, 2004; Cassani et al., 2018; Horvath et al., 2018; Babiloni et al., 2020a; Benwell et al., 2020; Wicki et al., 2021). Besides, AD is also characterized as a brain disconnection syndrome (Delbeuck et al., 2003). The synchronization of EEG activity is usually perturbed in AD patients, especially demonstrated by the decreased functional connectivity in different brain areas (Cassani et al., 2018; Musaeus et al.,2019a,b; Nunez et al., 2019; Song et al., 2019; Briels et al., 2020). Furthermore, the decline in the structure and functional connection of the brain may lead to a reduction in the complexity of EEG signals in AD patients, as reported in Azami et al. (2017), Deng et al. (2017), Kulkarni (2017), Simons and Abásolo (2017), Al-Nuaimi et al. (2018), Cassani et al. (2018), Horvath et al. (2018), Li et al. (2018), Simons et al. (2018). However, with respect to specific EEG frequency bands, current studies usually have inconsistent results, possibly due to multiple factors such as the severity level of disease, educational background of subjects, diagnostic criteria, sample sizes and the use of different computational measures (Briels et al., 2020). For example, Babiloni et al. found that subjective memory complaint seniors with AD neuropathology (amyloid PET-positive) and high education attainment showed higher temporal α3 power density and lower posterior α2 power density, suggesting that preclinical Alzheimer’s neuropathology may interact with education attainment (Babiloni et al., 2020b). Gaubert and colleagues found an increase in high frequency oscillations (higher β and γ power) and a decrease in low frequency oscillations (lower δ power), higher spectral entropy, higher complexity and increased functional connectivity in θ band in the frontocentral regions in preclinical AD patients (Gaubert et al., 2019). They found a nonlinear relationship between amyloid burden and EEG metrics in neurodegeneration positive subjects, suggesting the EEG patterns are modulated differently depending on the degree of amyloid burden. Briels and colleagues also found that the choice of functional connectivity measures and frequency bands can have a large impact on the outcome of EEG studies in AD. Their results showed that the corrected amplitude envelope correlation are reproducible in the α and β bands, and phase-based measures with correction for volume conduction showed reproducible effects in the θ band (Briels et al., 2020).

Considering the above inconsistent results, in this study we try to find significant and reliable EEG biomarkers for AD diagnosis. We collected 30 probable AD patients confirmed by PiB-PET inspection (AD group) with a wide neurological tests scores (MMSE and MoCA) range, resulting an AD population with great varieties of impaired cognitive functions. We calculated the power spectrum, spectral entropy and phase synchronization index metrics of their resting-state EEG in four frequency bands, and compared these metrics with that of 30 normal controlled elderly subjects (NC group). To investigate whether these EEG metrics could reflect the impaired cognitive functions in AD groups, we further calculated the correlation of these EEG metrics with the MMSE and MoCA test scores.

## Materials and Methods

### Participants

In this study, patients who were diagnosed clinically as probable AD were screened for inclusion from the outpatients in the Department of Neurology, the Second Medical Center of Chinese People’s Liberation Army General Hospital from 2016 to 2019. All patients in AD group meet the core criteria for probable AD diagnosis developed by the National Institute of Aging and the Alzheimer’s Disease Society (NIA-AA) in 2011 (McKhann et al., 2011). Besides, they all underwent the PiB-PET neuroimaging test and their results are all positive. At the same time, their family members and volunteers who matched their gender, age and education level were selected as normal controls (NC).

Thirty AD patients and 30 NC subjects were evaluated by two or more senior neurological physicians and were included in this study. The studies involving human participants were reviewed and approved by the Medical Ethics Committee of Chinese People’s Liberation Army General Hospital. The patients/participants provided their written informed consent to participate in this study.

### EEG Acquisition and Processing

#### EEG Acquisition

The EEG data acquisition of all the participants was completed in the fixed clinic of the Department of Neurology, Chinese People’s Liberation Army General Hospital. Before the test, the subjects were asked whether they took food or beverages containing stimulants such as nicotine, caffeine, and alcohol on the day, and the non-users were checked after washing their hair.

Participants sat in a comfortable chair, kept quiet and relaxed, and kept their bodies as motionless as possible to reduce artifacts. Because the EEG data collected when the eyes are open has more eye movement artifacts, the previous literature mostly uses the EEG data when the eyes are closed for research. Therefore, this study only records the resting state EEG data when the eyes are closed. The recording time is 5 min.

**FIGURE 1:**
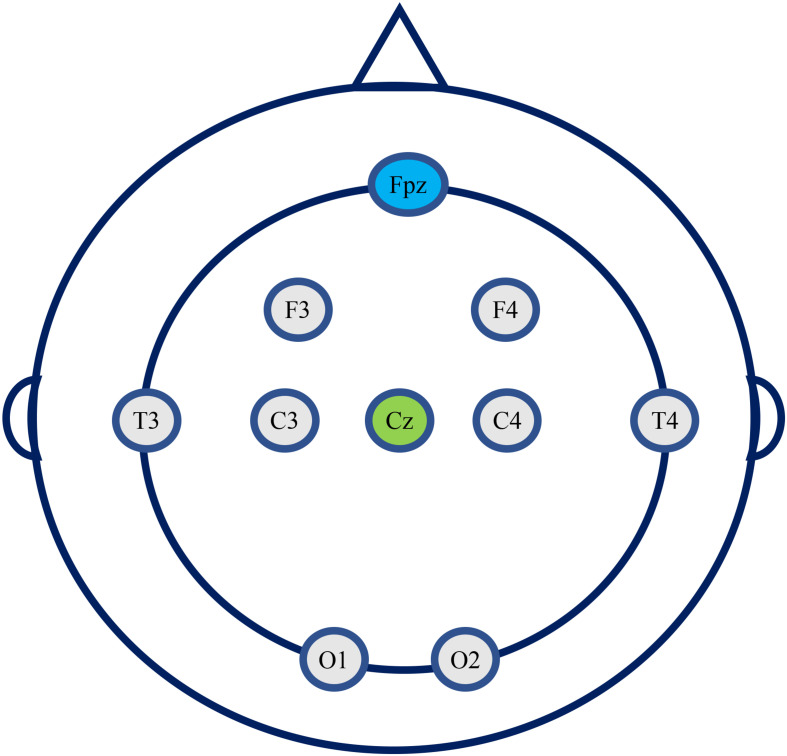
The eight electrodes position used in this study (Cz, reference electrode; Fpz, ground electrode).

The EEG detection equipment used is a portable 8-channel high-performance EEG signal acquisition instrument (JL-EEG8w), developed by the State Key Laboratory of Cognition and Learning of Beijing Normal University, which is designed for quick EEG examination from outpatients who usually have very limited time at the clinic. The bandwidth of the EEG amplifier is 0.1–80 Hz with a sampling frequency of 1,000 Hz. According to the 10–20 international standard electrode system, 8 electrode position is used, i.e., F3, F4, T3, T4, C3, C4, O1, and O2. The reference electrode is in Cz, and the ground electrode is in Fpz (Figure 1). There is no parietal electrode used due to the recording device’s limitation. The impedance of each active electrode is controlled below 100 kΩ before the start of recording EEG, and further checked after finishing recording. The data is saved in EDF format for subsequent offline analysis.

#### EEG Data Preprocessing

Several conventional preprocessing steps are taken following the recommendations from the OHBM COBIDAS MEEG committee (Pernet et al., 2020). Firstly, the recorded EEG data is bandpass filtered to 0.5–45 Hz, using the eegfiltfft function in EEGLab toolbox. Next, the continuous filtered data of each channel is divided into 4 s epoch. Then, for each epoch, the automatic artifact detection algorithm is applied to remove eye movement, breathing, EMG, 50 Hz power supply interference and outlier data segments, according to Durka et al. (2003). Finally, the remaining data epochs are manually checked to remove data segments that are not automatically eliminated with big artifacts or drowsy characteristics. The first 30 segments without artifacts (30 × 4 s = 120 s) of each channel of the participants were selected for analysis.

#### Relative Spectral Power of EEG

As mentioned in the above preprocessing section, the EEG signal has been divided into 30 epochs of 4 s data segment. We further reduce the sampling rate to 250 Hz, so that there are 1,000 points of EEG samples in each epoch. A Hamming window of 125 points (0.5 s) is used to slide over each piece of data in a step of 50 points (0.2 s). The Fourier transform of 1024 points is calculated to obtain the estimated power spectrum of each piece of data. The frequency resolution is about 0.25 Hz (Δf = Fs/Nfft = 250/1,024).

Then, the power spectrum estimates calculated for all 30 epochs are averaged, and the absolute powers are calculated in the following 4 canonical frequency bands: δ oscillation (1–4 Hz), θ oscillation (4–8 Hz), α oscillation (8–13 Hz), and β oscillation (13–30 Hz). In this study, we did not consider the γ oscillation as this frequency band EEG is easily contaminated by muscle artifacts.

Since the absolute power varies greatly among individual subjects, we further calculated the relative power of each oscillation for comparison between groups. Use the sum of the power in the frequency range of 1–45 Hz as the normalization factor, and define the ratio of each oscillation’s absolute powers to the normalization factor as the relative power of that oscillation. The pwelch function in Matlab software is used to estimate the power spectral density.

#### Spectral Entropy of EEG

In addition to the power spectrum characteristics of EEG signals, the complexity of EEG signals is also an important parameter reflecting the characteristics of EEG. EEG signals are generally considered to be a chaotic signal between random and deterministic signals. Entropy values can be used to quantitatively describe the uncertainty of EEG signals.

Spectral entropy refers to the degree of uncertainty of the signal power spectrum distribution. It regards the normalized power distribution of a signal in the frequency domain as a probability distribution, and then calculates its information entropy. For a signal x(n), its power spectrum is represented by S(ω), and the probability distribution p(ω) of the spectrum is defined as (Wang et al., 2015):

(1) $$p\,\left(\omega \right)=\frac{S\,\left(\omega \right)}{\sum_{i}S\,\left(i\right)}$$

Then the spectral entropy H is defined as:

(2) $$H\,=-\sum_{\omega =1}^{N}p\,\left(\omega \right)\,{\text{log}}_{2}\,p\,\left(\omega \right)$$

Here N is the total frequency point. Normalized spectral entropy is usually used and is defined as:

(3) $$H_{n}=-\frac{\sum_{m=1}^{N}P\,\left(m\right)\,l\,o\,g_{2}\,P\,\left(m\right)}{l\,o\,g_{2}\,N}$$

Here the denominator *log*_2_*N* represents the maximum spectral entropy of white noise evenly distributed in the frequency domain. The higher the spectral entropy of a signal, the more disordered (complex) the signal is. Conversely, the lower the spectral entropy, the more ordered (simple) the signal is.

In this study, the 8-s data sampled at 250 Hz is taken as one segment. There are a total of 15 segments. Calculate the spectral entropy in δ, θ, α, and β oscillation, and the frequency sampling points are 1,024 points. Then average the spectral entropy at each oscillation calculated from 15 data segments to obtain the spectral entropy value of each subject at each channel.

#### Phase Synchronization Index of EEG

Because brain information transmission needs to integrate the functions of various regions and the cooperation of neurons in multiple brain regions, the EEG in many diseases like AD are manifested as abnormal synchrony or connectivity between neurons in different brain regions. According to Dauwels et al. (2010), many of those synchronization measures are strongly correlated (or anti-correlated) with the correlation coefficient, providing little complementary information about EEG synchrony. While the phase synchrony indices are one of the metrics that are weakly correlated with the correlation coefficient, hence, it may capture a specific kind of interdependence of the EEG time series. The instantaneous phase φ_*x*_ of an EEG signal *x* is extracted as follows (Dauwels et al., 2010):

(4) $$\varphi_{x}\,\left(k\right)=\text{arg}\,\left[x\,\left(k\right)+i\,\tilde{x}\,\left(k\right)\right]$$

where $\tilde{x}$ is the Hilbert transform of *x*. The phase synchronization index is defined as:

(5) $$\gamma =\left|\left\langle e^{i\,\left(n\,\varphi_{x}-m\,\varphi_{y}\right)}\right\rangle \right|$$

where *n* and *m* are integers, ⟨⋅⟩ denotes time average. If γ tends to 1 in the above formula, the two signals are in phase synchronization, and if γ tends to 0, the phase difference of the two signals is randomly distributed.

In this study, phase synchronization index was used to investigate the connectivity difference in EEG signals between AD and NC groups. After pre-processing, the data is resampled to 250 Hz and divided into 4-s epochs. One-second Hamming window is used, with a 0.5-second sliding steps. The phase synchronization index of δ, θ, α, and β oscillation of each subject’s EEG is calculated.

### Neuropsychological Scale Evaluation

Two senior neurological physicians used MMSE and MoCA to evaluate the participants’ cognitive functions. The test environment was quiet and the test scale versions were uniform. Among 60 participants, three AD patients and 7 NC subjects failed to complete MMSE/MoCA assessment because of noncooperation.

### Statistical Analysis

Two-sample *t*-test was used to compare the age differences between AD and NC groups. Chi-square test was used to compare their gender differences. Using a two-sample *t*-test with false discovery rate (FDR) correction, the differences in the relative power, spectral entropy and phase synchronization index in δ, θ, α, and β oscillation of EEG signals between AD patients and NC subjects are compared. Further, in the AD patient group, Spearman correlation analysis with FDR correction was used to calculate the correlation between EEG metrics and the degree of cognitive impairment (2 neuropsychological evaluation scores). Subject’s age was used as a control factor to eliminate its effect on the results. *P* < 0.05 was considered statistically significant.

## Results

### Demographic Information and Neuropsychological Test Comparisons Between AD and NC Groups

As shown in Table 1, there was no statistical difference in age and gender between the AD and NC groups (*P* > 0.05), and there were statistically significant differences in the neuropsychological evaluation results (*P* < 0.001). The MMSE and MoCA scores in AD group were significantly lower than those in the NC group.

**TABLE 1:** Participants demographic information.

	AD (*n* = 30)	NC (*n* = 30)	*P* value
Age(year)	68.83 ± 10.18	64.43 ± 10.55	0.106
Male : Female	12:18	12:18	0.879
MMSE^*a*^	12.89 ± 9.98	29.39 ± 0.89	0.000
MoCA^*a*^	10.48 ± 7.90	28.22 ± 1.98	0.000

*^*a*^Three AD patients and 7 NC subjects failed to complete MMSE/MoCA assessment.*

### Sex Differences of EEG Metrics in AD or NC Groups

To evaluation the sex differences in each group, we further compared the relative spectral power, spectral entropy and phase synchronization indices between female (*n* = 18) and male (*n* = 12) participants in AD or NC groups. Two-sample *t*-test with FDR correction was used for the comparison. Results were shown in the Supplementary Materials (Supplementary Figure 1).

### Differences of EEG Metrics Between AD and NC Groups

#### Relative Spectral Power

**FIGURE 2:**
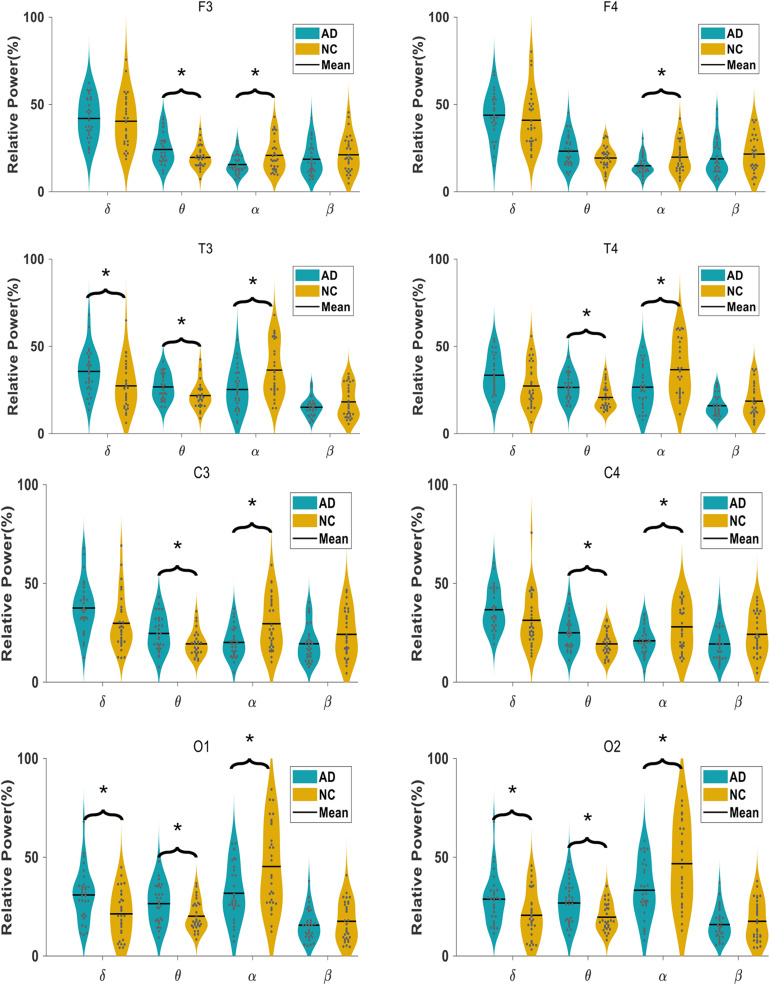
Comparison of relative spectral power in AD and NC group at each electrode in four frequency bands.

The comparison of the relative power of each oscillation at each electrode in the AD and NC groups is shown in Figure 2. The relative power of the slow wave oscillation (δ and θ oscillation) at each electrode in the AD group is higher than that in the NC group, and the relative power of fast-wave oscillation (α and β oscillation) at each electrode is lower than that in the NC group, suggesting that the relative power of EEG in AD patients is widely changed compared with NC subjects. Specifically, the relative spectral power of α oscillation at all electrodes and θ oscillation at seven electrodes (F3, T3, T4, C3, C4, O1, and O2) has significant difference, suggesting a diffuse slowing effect of the EEG spectrum in AD patients.

#### Spectral Entropy

**FIGURE 3:**
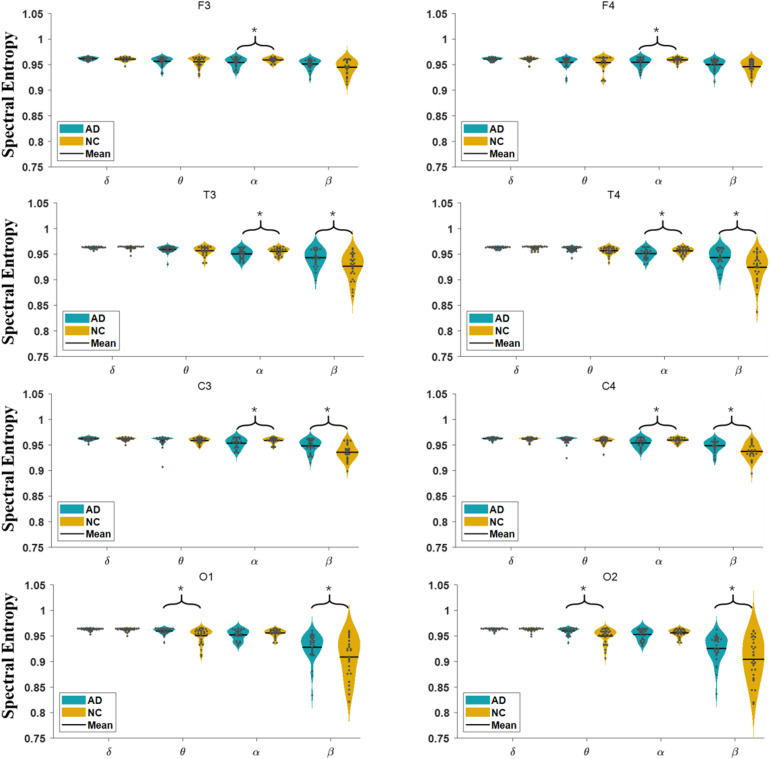
Comparison of spectral entropy in AD and NC group at each electrode in four frequency bands.

In Figure 3, we show the comparison of the spectral entropy of each oscillation at each electrode of the EEG in AD and NC groups. For the α oscillation, the spectral entropy in the frontal, temporal and central regions of the AD group is significantly decreased compared to NC group, while in the occipital electrodes the spectral entropy has no significant difference between groups. For the β oscillation, the spectral entropy in the temporal, central and occipital regions of the AD group is significantly higher than that of the NC group, but in the frontal area the spectral entropy has no significant difference between groups. The spectral entropy of the θ oscillation in the occipital areas of AD group was higher than that of the NC group. However, the spectral entropy in the δ oscillation does not show statistical difference between groups.

#### Phase Synchronization Index

**FIGURE 4:**
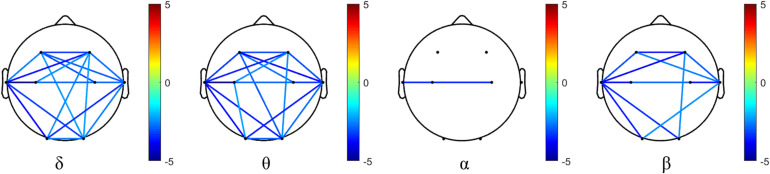
Differences of phase synchronization indices between AD and NC group in four frequency bands. Values beyond statistical significance after FDR correction is demonstrated, and the color bar denotes the *t*-value.

The phase synchronization comparison of each oscillation (δ, θ, α, and β) at each electrode pairs in the AD and NC groups is shown in Figure 4. The phase synchronization in AD group is significantly lower than that of the NC group, specifically in frontal and temporal related areas in δ, θ, and β oscillations. While for α oscillation, the phase synchronization index of AD groups in most areas (apart from T3 and C4 electrode pairs) does not show statistical significance.

### Association Between EEG Metrics and Neuropsychological Test Scores in AD Group

The Spearman correlation analysis with FDR correction was performed to investigate whether these EEG metrics could reflect the impaired cognitive functions in AD patients. Here we only demonstrate the association between EEG metrics and neuropsychological test scores in AD group. The neuropsychological tests and EEG correlation analysis in NC group and in both groups is listed in Supplementary Materials (Supplementary Tables 1–6).

#### Association Between Relative Spectral Power and MMSE/MoCA Scores

The correlation analysis between relative spectral power of each oscillation at each electrode of the EEG and the neuropsychological test scores in AD groups were calculated. As shown in Table 2, the relative power of δ and θ oscillations in AD patients is negatively correlated with the MMSE score, while the relative power of α and β oscillations is positively correlated with the MMSE score. Among them, the correlation of the relative power of α oscillation at bilateral frontal and central electrodes and the MMSE score is beyond statistical significance. The correlation between the power of each oscillation and the MoCA score has similar trend. The relative power of α oscillation at bilateral frontal-central and right occipital electrodes is significantly positively correlated with the MoCA score.

**TABLE 2:** Relationship between relative spectral power and MMSE/MoCA scores.

Electrode Position	MMSE				MoCA			
	ρ_*δ_ power*_	ρ_*θ_ power*_	ρ_*α_ power*_	ρ_*β_ power*_	ρ_*Ɵ_ power*_	ρ_*θ_ power*_	ρ_*α_ power*_	ρ_*β_ power*_
F3	0.0376	–0.3085	0.3736	0.2806	0.0523	–0.2197	0.4711	0.2207
F4	–0.1044	–0.2596	0.3267	0.4013	–0.0886	–0.1642	0.4369	0.3361
T3	–0.2575	–0.1636	0.6540** 0.1698	–0.2813	–0.2107	0.7103** 0.2199		
T4	–0.2596	–0.4019	0.5671* 0.4610	–0.2704	–0.3270	0.5871* 0.3999		
C3	–0.1034	–0.4856	0.5972* 0.3485	–0.0667	–0.4643	0.6320** 0.2894		
C4	–0.2275	–0.3480	0.5753* 0.0229	–0.2838	–0.3685	0.6470** 0.0838		
O1	–0.3660	–0.3205	0.4334	0.3962	–0.3380	–0.3951	0.4722	0.4280
O2	–0.4541	–0.2908	0.4717	0.3719	–0.3953* –0.3696	0.5315* 0.3902		

*P < 0.05; **P < 0.01.

#### Association Between Spectral Entropy and MMSE/MoCA Scores

We examined the correlation between δ, θ, α, and β oscillation spectrum entropy and the degree of cognitive impairment in the AD group. As shown in Table 3, the spectral entropy does not exhibit significant correlation to the MMSE and MoCA scores.

**TABLE 3:** Relationship between spectral entropy and MMSE/MoCA scores.

Electrode Position	MMSE				MoCA			
	ρ_*δ_ entropy*_	ρ_*θ_ entropy*_	ρ_*α_ entropy*_	ρ_*β_ entropy*_	ρ_*δ_ entropy*_	ρ_*θ_ entropy*_	ρ_*α_ entropy*_	ρ_*β_ entropy*_
F3	–0.3907	0.0542	0.3497	–0.2381	–0.2962	0.1014	0.3191	–0.1973
F4	–0.2999	0.0548	0.3027	0.0034	–0.2310	0.0440	0.2837	–0.0041
T3	–0.1264	0.2411	0.2387	–0.4739	–0.0734	0.1217	0.2947	–0.5415
T4	–0.2981	0.1387	0.3903	–0.3828	–0.2232	0.1133	0.3452	–0.4503
C3	–0.3528	0.0843	0.4075	–0.2785	–0.3027	0.0360	0.4055	–0.3572
C4	–0.4294	0.0854	0.2896	–0.5550	–0.3723	0.0434	0.3380	–0.5788
O1	–0.1919	0.1229	0.1812	–0.1962	–0.2445	0.0599	0.2598	–0.2075
O2	–0.1433	0.0294	0.1976	–0.1833	–0.2279	–0.0177	0.2609	–0.1996

#### Association Between Phase Synchronization and MMSE/MoCA Scores

We further investigated the association between δ, θ, α, and β oscillation phase synchronization indices and the degree of cognitive impairment in the AD group. From Table 4, we can see the phase synchronization index of β oscillation is significantly correlated with MoCA scores, specifically at left frontal-central and temporal-central electrode pairs. Besides, the correlation of phase synchronization index of θ oscillation at left central to right frontal and right temporal electrode pairs to MoCA scores also beyond statistical significance after FDR correction.

**TABLE 4:** Relationship between phase synchronization index and MMSE/MoCA scores.

Electrode pairs	MMSE				MoCA			
	ρ_*δ_ pha_ syn*_	ρ_*θ_ pha_ syn*_	ρ_*α_ pha_ syn*_	ρ_*β_ pha_ syn*_	ρ_*δ_ pha_ syn*_	ρ_*θ_ pha_ syn*_	ρ_*α_ pha_ syn*_	ρ_*β_ pha_ syn*_
F3-F4	0.0815	0.1249	–0.0084	0.3389	0.1377	0.1998	0.0650	0.4273
F3-T3	0.2913	0.2540	0.2156	0.4963	0.2882	0.3319	0.2902	0.5567*
F3-C3	0.0091	–0.0461	–0.0222	0.4796	–0.0025	–0.0057	0.0163	0.5542*
F3-C4	0.2166	0.2798	0.2513	0.4190	0.3530	0.3985	0.3314	0.5295
F3-T4	0.1624	0.4192	0.3108	0.4441	0.0978	0.4371	0.3564	0.5225
F3-O1	0.1683	0.3735	0.3115	0.4464	0.1610	0.4383	0.3258	0.5139
F3-O2	0.1017	0.2733	0.2059	0.4327	0.1030	0.3747	0.2548	0.5077
F4-T3	0.2546	0.2519	0.3283	0.3906	0.3551	0.3541	0.4125	0.4913
F4-C3	0.4255	0.5059	0.4528	0.4413	0.4387	0.5941*	0.4808	0.5127
F4-C4	–0.0245	0.0095	0.1032	0.0578	0.0641	0.0736	0.1711	0.1645
F4-T4	0.1470	0.2754	0.2773	0.2804	0.1856	0.2939	0.3197	0.3700
F4-O1	0.2665	0.2875	0.3547	0.4078	0.2822	0.3388	0.3568	0.4835
F4-O2	0.1943	0.1756	0.2995	0.3473	0.2252	0.2208	0.3278	0.4240
T3-C3	0.3450	0.3806	0.2854	0.4770	0.4309	0.4585	0.3698	0.6104*
T3-C4	0.3059	0.3649	0.4134	0.4915	0.3630	0.4391	0.4683	0.5555*
T3-T4	0.1137	0.3751	0.3269	0.3407	0.0761	0.3285	0.2606	0.3260
T3-O1	0.0124	0.2274	0.2194	0.2123	0.0076	0.1793	0.1252	0.2145
T3-O2	0.0426	0.1717	0.1869	0.2107	–0.0014	0.1135	0.0591	0.1840
C3-C4	0.4031	0.3382	0.3449	0.4251	0.4693	0.4209	0.3630	0.5261
C3-T4	0.3343	0.5681	0.4578	0.5410	0.3246	0.5836*	0.4782	0.6553*
C3-O1	0.3655	0.3330	0.2635	0.4735	0.3362	0.3667	0.2670	0.5600*
C3-O2	0.1847	0.2222	0.2591	0.3949	0.1630	0.2534	0.2691	0.5277
C4-T4	–0.0414	0.1907	0.2125	0.3301	0.0520	0.3367	0.3223	0.4557
C4-O1	0.2332	0.2900	0.3895	0.3570	0.2160	0.3039	0.3517	0.4109
C4-O2	0.2106	0.2772	0.2908	0.2874	0.1481	0.2765	0.2575	0.3198
T4-O1	0.0493	0.2618	0.1802	0.2722	0.0244	0.1878	0.0611	0.2347
T4-O2	–0.0439	0.2368	0.2054	0.2378	–0.0782	0.1744	0.1074	0.2288
O1-O2	–0.0826	–0.0150	-0.0980	0.1018	–0.1033	–0.0979	–0.2208	0.0197

*P < 0.05.

## Discussion

In this study, we found that, almost in the whole brain regions, the AD group had higher θ oscillation spectral power and lower α oscillation spectral power than that in the NC group, which is consistent with many former studies as reviewed in Jeong (2004); Engels et al. (2017), Malek et al. (2017); Cassani et al. (2018), Horvath et al. (2018), and Babiloni et al. (2020a). We further found that in the AD group the α oscillation spectral power was positively correlated with the MMSE and MoCA scores. Basar and colleagues has suggested α oscillations have multifold functional correlates including sensory, motor and memory functions (Basar and Guntekin, 2012). As a universal code or universal operator, α oscillations serve as building blocks in several functions and can be used as clinical biomarkers of cognitive impairment in schizophrenia, Alzheimer’s disease and bipolar disorders (Basar and Guntekin, 2012). Our results showed that α oscillation spectral power could be a significant EEG biomarker for differentiating probable AD patients from normal elderly, and could also indirectly reflect the severity and prognosis of disease.

Previous studies have shown that the EEG complexity of AD patients is lower than that of NC subjects, which is manifested by changes in entropy-related parameters such as spectral entropy, approximate entropy, and sample entropy (Abasolo et al., 2006; Simons et al., 2018; Tylová et al., 2018), etc. While on different time scales or frequency bands, the complexity of EEG signals associated with cognitive impairment may be inconsistent. When multi-scale entropy analysis is used, the entropy in the AD group was lower than that in the NC group on smaller scales, while the AD patients had higher complexity than NC subjects at larger scales on long scales (Mizuno et al., 2010; Maturana-Candelas et al., 2019). The smaller/larger scales can be considered to correspond to higher/lower frequencies of spectral power, respectively. Spectral entropy considers the complexity at specific frequency bands. Our study found that the spectral entropy of the α oscillations in the frontal, temporal and central regions of the AD group was lower than that in the NC group, which is consistent with former studies (Sun et al., 2020). On the other hand, we found the β oscillation spectral entropy in the temporal, central and occipital regions was higher than that in the NC group. Because the spectral entropy represents the degree of uncertainty in the power distribution of EEG signals, our results suggest that the power fluctuations of the α oscillations of AD patients become smaller, while the power fluctuations of the β oscillations become larger. In preclinical amyloid positive patients, Gaubert and colleagues also found increased spectral entropy, which is suggested to be related to a compensatory mechanism in AD patients during memory load and cognitive performance (Gaubert et al., 2019).

Many functional connectivity measures has been used in evaluating the synchronization characteristics in different brain areas in AD patients, and many of them are correlated (Dauwels et al., 2010). The phase-based measures are robust and reproducible, insensitive to volume conduction (Briels et al., 2020). Our study found the phase synchronization index of δ, θ, and β oscillations of AD patients was decreased than that of the NC subjects, especially in the frontal, temporal and central areas. Further, we found the phase synchronization index of β oscillations at these brain areas correlated well with the MoCA scores in AD patients. According to Pini et al. (2016), cortical atrophy in AD patients affects the medial temporal lobe very early, then extending to the other parts of the cortex along a temporal-parietal-frontal trajectory. Due to the recording device’s limitation, we lack the neural activity in the parietal electrodes. Instead, our results suggests the neuronal functional connectivity at the temporal-central-frontal areas in AD patients is greatly impaired, and the phase synchronization index of β oscillations might be an indicator of the impairment of brain functions. However, the phase synchronization index of α oscillation seemed less damaged and less correlated with the neurological scores, suggesting the phase synchronization feature of α oscillations may not reflect the severity of disease.

In conclusion, our study suggest that quantitative EEG spectral power in α oscillations and phase synchronization characteristics in β oscillations could reflect the severity of AD disease and are beneficial to the diagnosis and screening of probable AD patients. As the PiB-PET examination is rather expensive, the number of AD patients included in this study is relatively small. In addition, the parietal neural activity is not recorded in current study, which hindered us evaluating the impaired cognitive functions in parietal areas of AD patients. Further, this study is a retrospective, cross-sectional group study. In the future we should consider a longitudinal and individualized study with a larger sample size and with more electrode sites.

## Data Availability Statement

The data that support the findings of this study are available from the corresponding authors, upon reasonable request.

## Ethics Statement

The studies involving human participants were reviewed and approved by Medical Ethics Committee of Chinese People’s Liberation Army General Hospital. The patients/participants provided their written informed consent to participate in this study.

## Author Contributions

YW, SZ, YG, WD, YG, LL, and MS recruited the participants and collected their information. HZ carried out the statistical analysis. XG collected and processed the EEG signals. FY interpreted the results. XL and LW designed the study. HZ, XG, and FY wrote the manuscript. All authors reviewed the manuscript.

## Conflict of Interest

The authors declare that the research was conducted in the absence of any commercial or financial relationships that could be construed as a potential conflict of interest.
