# Supplementary material for: The Significance of EEG Alpha Oscillation Spectral Power and Beta Oscillation Phase Synchronization for Diagnosing Probable Alzheimer Disease
Source: Front Aging Neurosci. 2021 Jun 7;13:631587. doi: 10.3389/fnagi.2021.631587 (PMC8215164; doi:10.3389/fnagi.2021.631587)
Supplement: Supplementary file 1 [file Table_1.docx]

**Supplementary Materials**

**Sex Differences of EEG metrics in AD or NC groups**

we compared the relative spectral power/spectral entropy/phase synchronization indics between female (n=18) and male (n=12) participants in AD or NC groups. Two-sample t test with false discovery rate (FDR) correction was used for the comparison.

For the relative spectral power and spectral entropy in 4 frequency bands at all 8 electrodes, we found no difference between the female and male participants in AD or NC groups (figurs not shown).

As for the phase synchronization indices in 4 frequency bands at all electrode pairs, we found no difference between the female and male participants in the NC group (figurs not shown).

While in the AD group, female participants demonstrate higher α oscillation phase synchronization indices compared to male participants in right central to right occipital electrode pairs (C4-O2, P=0.0108), as shown in Fig. 1. We thought this might because the female AD patients are less damaged in the right central to right occipital areas compared to male AD patients.


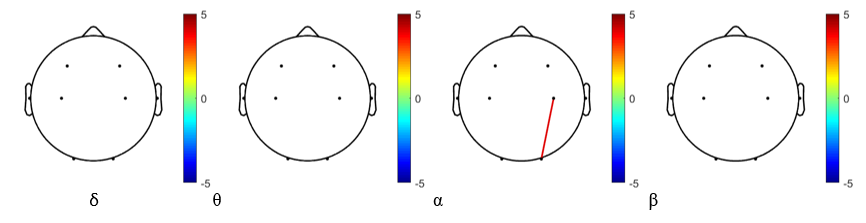


Fig. S1 The comparison of phase synchronization indices in female and male participants in AD groups in four frequency bands. Values beyond statistical significance after FDR correction is demonstrated, and the color bar denotes the t-value.

**Association between EEG metrics and neuropsychological test scores in NC group and in both groups**

We have calculated the Spearman correlation analysis after FDR correction of 3 EEG metrics and 2 neuropsychological tests scores for NC group and for both groups. We listed the correlation values below.

Because the subjects in NC group almost got full scores in these two neuropsychological tests, these EEG metrics (relative spectral power, spectral entropy, phase synchronization indices) in almost whole brain areas (apart from the spectral entropy in occipital areas) are not significantly related to the MMSE/MoCA scores.

As for both groups, similar to the results in AD group, the relative spectral power of delta and theta oscillation are significantly negatively related to the MMSE/MoCA scores, and the relative spectral power of alpha and beta oscillation are significantly positively related to the MMSE/MoCA scores. Especially for the alpha oscillation spectral power, the statistical value P is less than 0.01 at all electrodes, which further support the main result in our manuscript that alpha power is a significant metric for diagnosing/screening probable AD.

The beta oscillation spectral entropy also demonstrates significant correlation to the MMSE/MoCA scores in both groups. However, considering the spectral entropy metrics are not related to these scores in AD group, we hesitate to say that spectral entropy of beta oscillation is a significant metric for diagnosing probable AD.

Similar to our results in AD group, the phase synchronization indices for both groups in right frontal/temporal/central related areas at 4 frequency bands are significantly related to these two test scores. Considering in the AD group, only the beta oscillation phase synchronization indices at left frontal-central and temporal-central electrodes are beyond statistical threshold, we think the beta oscillation phase synchronization indices in these areas are significant metrics for diagnosing/screening probable AD.

**Table S1 Relationship between relative spectral power and MMSE/MoCA scores in NC group**

| Electrode  Position | MMSE | | | | MoCA | | | |
| --- | --- | --- | --- | --- | --- | --- | --- | --- |
|  | $\rho_{\delta\_power}$ | $\rho_{\theta\_power}$ | $\rho_{\alpha\_power}$ | $\rho_{\beta\_power}$ | $\rho_{\delta\_power}$ | $\rho_{\theta\_power}$ | $\rho_{\alpha\_power}$ | $\rho_{\beta\_power}$ |
| F3 | -0.3243 | -0.0609 | 0.2699 | 0.1846 | -0.2401 | -0.0284 | 0.3277 | 0.0912 |
| F4 | -0.1697 | -0.1369 | 0.2190 | -0.0132 | -0.0964 | -0.1942 | 0.2842 | -0.2082 |
| T3 | -0.4518 | -0.2953 | 0.3719 | 0.0551 | -0.2895 | -0.2969 | 0.4033 | 0.0308 |
| T4 | -0.3337 | -0.0427 | 0.3188 | -0.0945 | -0.2797 | -0.0641 | 0.3381 | -0.1334 |
| C3 | -0.3503 | -0.0662 | 0.3367 | 0.0352 | -0.2381 | -0.0164 | 0.3722 | -0.0926 |
| C4 | -0.4886 | -0.3273 | 0.4611 | -0.0443 | -0.2782 | -0.3370 | 0.3931 | 0.0054 |
| O1 | -0.4467 | -0.3562 | 0.4085 | -0.2798 | -0.4305 | -0.4154 | 0.4041 | -0.2051 |
| O2 | -0.4276 | -0.3678 | 0.3939 | -0.2110 | -0.3743 | -0.4294 | 0.4275 | -0.1420 |

**Table S2 Relationship between spectral entropy and MMSE/MoCA scores in NC group**

| Electrode  Position | MMSE | | | | MoCA | | | |
| --- | --- | --- | --- | --- | --- | --- | --- | --- |
|  | $\rho_{\delta\_entropy}$ | $\rho_{\theta\_entropy}$ | $\rho_{\alpha\_entropy}$ | $\rho_{\beta\_entropy}$ | $\rho_{\delta\_entropy}$ | $\rho_{\theta\_entropy}$ | $\rho_{\alpha\_entropy}$ | $\rho_{\beta\_entropy}$ |
| F3 | 0.3094 | 0.3701 | 0.3767 | -0.2117 | 0.1428 | 0.2189 | 0.1145 | -0.1297 |
| F4 | 0.1899 | 0.2315 | 0.2396 | -0.2101 | 0.0770 | 0.0970 | 0.0205 | -0.1850 |
| T3 | 0.3368 | -0.4775 | 0.2147 | -0.1978 | 0.0610 | -0.4524 | 0.1054 | -0.3870 |
| T4 | 0.2986 | -0.0993 | 0.0600 | -0.3924 | 0.0268 | -0.0228 | -0.1297 | -0.4310 |
| C3 | 0.4955 | 0.0070 | 0.0989 | -0.3169 | 0.3324 | 0.1316 | -0.1270 | -0.2126 |
| C4 | 0.2326 | -0.6060* | 0.1480 | -0.2379 | 0.0826 | -0.4733 | 0.0640 | -0.2754 |
| O1 | 0.3505 | -0.6256* | 0.1337 | -0.4288 | 0.1331 | -0.6269* | 0.0853 | -0.5226 |
| O2 | 0.2758 | -0.6423* | 0.1208 | -0.4417 | 0.0558 | -0.6127* | 0.0580 | -0.4573 |

Note: *P<0.05

**Table S3 Relationship between phase synchronization index and MMSE/MoCA scores in NC group**

| Electrode  Pairs | MMSE | | | | MoCA | | | |
| --- | --- | --- | --- | --- | --- | --- | --- | --- |
|  | $\rho_{\delta\_pha\_syn}$ | $\rho_{\theta\_pha\_syn}$ | $\rho_{\alpha\_pha\_syn}$ | $\rho_{\beta\_pha\_syn}$ | $\rho_{\delta\_pha\_syn}$ | $\rho_{\theta\_pha\_syn}$ | $\rho_{\alpha\_pha\_syn}$ | $\rho_{\beta\_pha\_syn}$ |
| F3-F4 | -0.0265 | 0.0406 | 0.0106 | -0.1468 | 0.1434 | 0.0505 | -0.0203 | -0.0993 |
| F3-T3 | -0.1333 | -0.1968 | -0.2281 | -0.3759 | 0.3928 | 0.2651 | 0.1806 | 0.0056 |
| F3-C3 | 0.0391 | 0.2091 | 0.2900 | -0.1646 | 0.0178 | 0.0133 | 0.0786 | -0.2476 |
| F3-C4 | -0.0771 | -0.0546 | -0.1339 | -0.2726 | -0.0959 | -0.1194 | -0.1111 | -0.3322 |
| F3-T4 | -0.0587 | -0.0322 | -0.1364 | -0.1926 | 0.2433 | 0.1152 | -0.0501 | -0.1090 |
| F3-O1 | -0.0950 | -0.2284 | -0.1958 | -0.3172 | 0.2601 | -0.0928 | 0.0450 | -0.3755 |
| F3-O2 | -0.2018 | -0.2466 | -0.3417 | -0.3297 | 0.0972 | -0.1565 | -0.1298 | -0.3064 |
| F4-T3 | -0.2054 | -0.0908 | -0.1385 | -0.2701 | 0.3411 | 0.2200 | 0.2286 | 0.0010 |
| F4-C3 | -0.2568 | -0.0761 | -0.1074 | -0.2705 | 0.0340 | -0.0373 | -0.2191 | -0.2959 |
| F4-C4 | 0.0947 | 0.0352 | 0.0681 | -0.1405 | 0.0783 | 0.0397 | 0.0690 | -0.1380 |
| F4-T4 | -0.0057 | 0.0402 | -0.0148 | -0.1857 | 0.1432 | -0.0279 | -0.1056 | -0.1057 |
| F4-O1 | -0.1870 | -0.1883 | -0.3339 | -0.3764 | 0.1657 | -0.1772 | -0.1767 | -0.2819 |
| F4-O2 | -0.3315 | -0.3347 | -0.3319 | -0.4278 | 0.0631 | -0.3453 | -0.3255 | -0.3175 |
| T3-C3 | 0.1856 | 0.1448 | 0.0372 | -0.0918 | 0.1772 | 0.1319 | 0.1111 | 0.1053 |
| T3-C4 | 0.2062 | 0.1068 | -0.0403 | -0.1465 | 0.3943 | 0.2104 | 0.1429 | -0.0672 |
| T3-T4 | -0.1699 | -0.1768 | -0.2450 | -0.2454 | 0.2054 | 0.2402 | 0.0736 | 0.0224 |
| T3-O1 | 0.1393 | -0.0218 | -0.1458 | -0.1521 | -0.1211 | -0.0797 | 0.0569 | -0.1543 |
| T3-O2 | 0.1876 | -0.1020 | -0.0862 | -0.1095 | 0.0288 | 0.0068 | 0.1258 | -0.0027 |
| C3-C4 | -0.1565 | -0.3129 | -0.3806 | -0.2154 | 0.1363 | -0.1255 | -0.2142 | -0.2017 |
| C3-T4 | 0.0496 | 0.0325 | -0.1071 | -0.2277 | 0.1811 | 0.0461 | -0.0860 | -0.1819 |
| C3-O1 | 0.0065 | 0.0030 | -0.1512 | -0.2841 | 0.1651 | 0.0236 | 0.0709 | -0.1485 |
| C3-O2 | 0.0324 | -0.1487 | -0.1808 | -0.2542 | 0.1436 | -0.0886 | -0.0296 | -0.1434 |
| C4-T4 | 0.2761 | 0.1379 | -0.0399 | 0.0165 | 0.2545 | 0.0333 | -0.0417 | -0.0710 |
| C4-O1 | -0.0178 | -0.1548 | -0.1987 | -0.2083 | 0.3047 | -0.0352 | 0.0394 | -0.1459 |
| C4-O2 | -0.1122 | -0.2554 | -0.3192 | -0.2583 | 0.1443 | -0.0568 | -0.1552 | -0.2197 |
| T4-O1 | -0.0544 | -0.1862 | -0.1679 | -0.2370 | -0.1599 | -0.3370 | -0.2294 | -0.3688 |
| T4-O2 | -0.0877 | -0.1191 | -0.0772 | -0.2252 | -0.2662 | -0.3141 | -0.2634 | -0.3260 |
| O1-O2 | -0.0902 | -0.1218 | -0.2002 | -0.2278 | -0.1005 | -0.0856 | 0.0242 | -0.0809 |

**Table S4 Relationship between relative spectral power and MMSE/MoCA scores in both groups**

| Electrode  Position | MMSE | | | | MoCA | | | |
| --- | --- | --- | --- | --- | --- | --- | --- | --- |
|  | $\rho_{\delta\_power}$ | $\rho_{\theta\_power}$ | $\rho_{\alpha\_power}$ | $\rho_{\beta\_power}$ | $\rho_{\delta\_power}$ | $\rho_{\theta\_power}$ | $\rho_{\alpha\_power}$ | $\rho_{\beta\_power}$ |
| F3 | -0.2380 | -0.3330* | 0.4937** | 0.3291* | -0.2123 | -0.3193* | 0.5322** | 0.2762 |
| F4 | -0.2168 | -0.3018* | 0.4422** | 0.3255* | -0.1926 | -0.3021* | 0.4764** | 0.2613 |
| T3 | -0.5466** | -0.3999** | 0.6152** | 0.1277 | -0.5498** | -0.3990** | 0.6511** | 0.1217 |
| T4 | -0.4969** | -0.3802* | 0.6020** | 0.3761* | -0.4969** | -0.3648 | 0.6295** | 0.3375* |
| C3 | -0.4172** | -0.4514** | 0.5211** | 0.3897** | -0.3992** | -0.4441** | 0.5486** | 0.3512* |
| C4 | -0.5212** | -0.4792** | 0.5763** | 0.1101 | -0.5314** | -0.4867** | 0.6063** | 0.1403 |
| O1 | -0.5116** | -0.4452** | 0.5129** | 0.0726 | -0.5126** | -0.4771** | 0.5288** | 0.0650 |
| O2 | -0.4889** | -0.4505** | 0.4886** | 0.0414 | -0.4640** | -0.4817** | 0.5106** | 0.0272 |

Note: *P<0.05, **P<0.01

**Table S5 Relationship between spectral entropy and MMSE/MoCA scores in both groups**

| Electrode  Position | MMSE | | | | MoCA | | | |
| --- | --- | --- | --- | --- | --- | --- | --- | --- |
|  | $\rho_{\delta\_entropy}$ | $\rho_{\theta\_entropy}$ | $\rho_{\alpha\_entropy}$ | $\rho_{\beta\_entropy}$ | $\rho_{\delta\_entropy}$ | $\rho_{\theta\_entropy}$ | $\rho_{\alpha\_entropy}$ | $\rho_{\beta\_entropy}$ |
| F3 | -0.1740 | 0.1765 | 0.4023* | -0.3331* | -0.1857 | 0.1540 | 0.3499* | -0.3293* |
| F4 | -0.1244 | 0.2065 | 0.3657* | -0.1809 | -0.1276 | 0.1755 | 0.3332* | -0.1902 |
| T3 | 0.1408 | -0.2652 | 0.3161 | -0.4854** | 0.1265 | -0.2867 | 0.3032 | -0.5580** |
| T4 | -0.0795 | -0.0262 | 0.3620* | -0.5459** | -0.1079 | -0.0463 | 0.3295* | -0.5927** |
| C3 | -0.1748 | -0.1789 | 0.4198* | -0.4876** | -0.1963 | -0.1912 | 0.3814* | -0.5302** |
| C4 | -0.0017 | -0.3724 | 0.3114 | -0.5265** | -0.0019 | -0.3633* | 0.3100 | -0.5515** |
| O1 | -0.0917 | -0.4816** | 0.1176 | -0.3440* | -0.1558 | -0.5030** | 0.1265 | -0.3922* |
| O2 | -0.1351 | -0.5453** | 0.0855 | -0.3528* | -0.2228 | -0.5522** | 0.0834 | -0.3952* |

Note: *P<0.05, **P<0.01

**Table S6 Relationship between phase synchronization index and MMSE/MoCA scores in both groups**

| Electrode  Pairs | MMSE | | | | MoCA | | | |
| --- | --- | --- | --- | --- | --- | --- | --- | --- |
|  | $\rho_{\delta\_pha\_syn}$ | $\rho_{\theta\_pha\_syn}$ | $\rho_{\alpha\_pha\_syn}$ | $\rho_{\beta\_pha\_syn}$ | $\rho_{\delta\_pha\_syn}$ | $\rho_{\theta\_pha\_syn}$ | $\rho_{\alpha\_pha\_syn}$ | $\rho_{\beta\_pha\_syn}$ |
| F3-F4 | 0.2249 | 0.2815 | 0.2172 | 0.3537* | 0.2760 | 0.2747 | 0.2110 | 0.3713* |
| F3-T3 | 0.2153 | 0.2447 | 0.1638 | 0.2983 | 0.2886 | 0.3099* | 0.2349 | 0.3445* |
| F3-C3 | 0.3611* | 0.3662* | 0.3653* | 0.4942* | 0.3727* | 0.3395* | 0.3329* | 0.4934* |
| F3-C4 | 0.1059 | 0.2123 | 0.1093 | 0.2282 | 0.1488 | 0.2161 | 0.1280 | 0.2449 |
| F3-T4 | 0.3961* | 0.4700* | 0.3597* | 0.3924* | 0.4156* | 0.4740* | 0.3586* | 0.3955* |
| F3-O1 | 0.4000* | 0.3436* | 0.2754 | 0.3609* | 0.4534* | 0.3629* | 0.3140* | 0.3541* |
| F3-O2 | 0.3897* | 0.3747* | 0.2947 | 0.3482* | 0.4456* | 0.4017* | 0.3499* | 0.3531* |
| F4-T3 | 0.1072 | 0.1490 | 0.1098 | 0.1862 | 0.2259 | 0.2069 | 0.1861 | 0.2490 |
| F4-C3 | 0.2475 | 0.3409* | 0.3373 | 0.3128 | 0.3002* | 0.3494* | 0.3105* | 0.3158* |
| F4-C4 | 0.1141 | 0.1684 | 0.2239 | 0.1421 | 0.1406 | 0.1733 | 0.2395 | 0.1646 |
| F4-T4 | 0.1966 | 0.2100 | 0.1624 | 0.2525 | 0.2111 | 0.1690 | 0.1304 | 0.2690 |
| F4-O1 | 0.2267 | 0.2281 | 0.1614 | 0.2484 | 0.3091* | 0.2258 | 0.1722 | 0.2632 |
| F4-O2 | 0.2783 | 0.1841 | 0.2026 | 0.2615 | 0.3497* | 0.1767 | 0.1992 | 0.2879 |
| T3-C3 | 0.2917 | 0.3259* | 0.2859 | 0.2765 | 0.3102* | 0.3304* | 0.3147 | 0.3363* |
| T3-C4 | 0.0294 | 0.1944 | 0.1842 | 0.1405 | 0.0848 | 0.2364 | 0.2215 | 0.1635 |
| T3-T4 | 0.3553* | 0.4044* | 0.2245 | 0.3020 | 0.3844* | 0.4390* | 0.2320 | 0.3050* |
| T3-O1 | 0.2803 | 0.2534 | 0.0686 | 0.1857 | 0.2547 | 0.2446 | 0.0886 | 0.1814 |
| T3-O2 | 0.2656 | 0.1867 | 0.0710 | 0.2177 | 0.2445 | 0.1877 | 0.0872 | 0.2230 |
| C3-C4 | 0.0607 | 0.1697 | 0.2166 | 0.1728 | 0.1413 | 0.2146 | 0.2643 | 0.2121 |
| C3-T4 | 0.4056* | 0.4494* | 0.3823 | 0.4332* | 0.4182* | 0.4447* | 0.3729* | 0.4477* |
| C3-O1 | 0.3290* | 0.3071 | 0.2641 | 0.3416* | 0.3457* | 0.3208* | 0.3120* | 0.3893* |
| C3-O2 | 0.4225* | 0.2891 | 0.3014 | 0.4107* | 0.4274* | 0.3164* | 0.3484* | 0.4570* |
| C4-T4 | 0.2026 | 0.2924 | 0.2976 | 0.3046 | 0.2291 | 0.2874 | 0.3012 | 0.3128 |
| C4-O1 | 0.1607 | 0.1358 | 0.0807 | 0.1366 | 0.2076 | 0.1554 | 0.1146 | 0.1597 |
| C4-O2 | 0.1707 | 0.2074 | 0.1847 | 0.2274 | 0.2066 | 0.2394 | 0.2142 | 0.2473 |
| T4-O1 | 0.2904 | 0.2522 | 0.0687 | 0.2298 | 0.2562 | 0.1791 | 0.0163 | 0.1823 |
| T4-O2 | 0.2094 | 0.2044 | 0.1114 | 0.2296 | 0.1675 | 0.1434 | 0.0493 | 0.1959 |
| O1-O2 | 0.1471 | 0.0807 | -0.1237 | 0.0951 | 0.1316 | 0.0644 | -0.1056 | 0.0954 |

Note: *P<0.05
